# The Effects of a High Magnetic Field on the Annealing of [(Fe_0.5_Co_0.5_)_0.75_B_0.2_Si_0.05_]_96_Nb_4_ Bulk Metallic Glass

**DOI:** 10.3390/ma9110899

**Published:** 2016-11-04

**Authors:** Peng Jia, En-gang Wang, Ke Han

**Affiliations:** 1 Key Laboratory of Electromagnetic Processing of Materials (Ministry of Education), Northeastern University, Shenyang 110819, China; pjia@epm.neu.edu.cn; 2 National High Magnetic Field Laboratory, Florida State University, Tallahassee, FL 32310, USA; han@magnet.fsu.edu

**Keywords:** bulk metallic glasses, soft magnetic material, nanocrystalline composite, high magnetic field, crystallization

## Abstract

In contrast with amorphous alloys, nanocrystalline soft magnetic materials show improved thermal stability and higher soft magnetic properties. The nanocrystalline soft magnetic composites are usually fabricated by partially crystallizing from parent amorphous alloys. This paper reports our experimental observation on the sequence of crystallization in metallic glass under a high magnetic field (HMF). An application of a HMF to bulk metallic glass (BMG) of [(Fe_0.5_Co_0.5_)_0.75_B_0.2_Si_0.05_]_96_Nb_4_ prioritizes the precipitation of α-(Fe,Co) phase separated from the subsequent precipitation of borides, (Fe,Co)_23_B_6_, upon isothermal annealing at a glass transition temperature. Furthermore, it was observed that, through the annealing treatment under a HMF, a soft magnetic nanocomposite, in which only α-(Fe,Co) phase uniformly distributes in amorphous matrix, was achieved for boron-bearing BMG. The promotion of the α-Fe or (Fe,Co) phase and the prevention of the boride phases during the isothermal annealing process help to produce high-quality soft magnetic nanocomposite materials. The mechanism by which a HMF influences the crystallization sequence was interpreted via certain changes in Gibbs free energies for two ferromagnetic phases. This finding evidences that the annealing treatment under a HMF is suitable for enhancing the soft magnetic properties of high B content (Fe,Co)-based bulk amorphous and nanocrystalline materials.

## 1. Introduction

Soft magnetic materials with low core losses, high magnetization, and low cost are the key components for transformers with improved energy efficiency, especially in higher frequency and elevated temperature operation conditions [1,2]. Since the 1970s, greatly reduced core loss has been achieved in amorphous and nanocrystalline alloys [3,4,5,6,7,8]. Because of a lack of magnetic domain walls, the soft magnetic properties of amorphous alloys deteriorate rapidly under operation frequency from tens to hundreds of kilohertz. In contrast with amorphous alloys, nanocrystalline soft magnetic materials show improved thermal stability, higher magnetization, and lower core loss [6,7,8]. The remarkable reduction in coercivity with decreasing grain size was experimentally observed and explained by Herzer [9] with the assumption that the randomly oriented grains are sufficiently exchange-coupled through the intergranular amorphous matrix. The nanocrystalline soft magnetic materials were usually fabricated via partially crystallization from parent amorphous alloys. Upon heating, a large number of randomly oriented nanocrystalline phases (usually α-Fe, Fe_3_Si, or α- or α’-(Fe,Co) phase with cubic symmetry) have been formed in the amorphous matrix at a primary crystallization temperature (*T*_x1_) [6,7]. The composition variation in the amorphous phase and exchange-coupling between nanocrystalline phase and amorphous phase resulted in an increase in Curie temperature [10,11,12]. However, at higher crystallization temperatures, intermetallic phases (e.g., Fe_23_Zr_6_, Fe_23_B_6_, Fe_2_Zr, and Fe_2_B phases [10,11,12,13]) form from the amorphous phase, leading to the grain coarsening and deterioration of properties. Therefore, the nanocrystalline phases with cubic symmetry are desirable without formation of other intermetallic phases. For these reasons, the conventional nanocrystalline (Fe,Co)-based soft magnetic materials have to be featured with (i) relatively low B content (<15 atom %); (ii) the additions of small amounts of Cu or Au, which favors clustering during early stages of annealing, thus providing a multitude of heterogeneous nucleation sites for the nanocrystallites; (iii) the addition of a small amount of early transition metals (e.g., Nb, Zr and Hf), which limits the diffusivity of Fe atoms, thereby inhibiting grain coarsening of the nanocrystallites. The conventional alloying method for the production of soft magnetic nanocomposites limits the composition design of the alloy. Too much non-ferromagnetic elements decrease the alloy saturation magnetization. High B content (>20 atom %), which is preferred in alloys with high glass forming ability (GFA), usually leads to the formation of boride such as Fe_23_B_6_, Fe_2_B, and Fe_3_B phases in the primary crystallization and low density of α-Fe phase, which deteriorates the soft magnetic properties [14]. Thus, the dilemma between the GFA and magnetic properties has stimulated a considerable amount of research activity.

Recently, high magnetic fields (HMFs) have been successfully applied to materials design and preparation [15,16,17,18,19]. Results have demonstrated that the magnetic field is a powerful tool to affect the crystallization process of metallic glasses and the texture formation of the crystallized phases [20,21,22,23]. In this work, HMFs were introduced in the annealing process of a [(Fe_0.5_Co_0.5_)_0.75_B_0.2_Si_0.05_]_96_Nb_4_ bulk metallic glass (BMG) alloy with high boron content. We found that the crystallization sequence during the primary crystallization process was changed for α-(Fe,Co) and (Fe,Co)_23_B_6_ phases due to HMFs, and their coexistence during the primary crystallization process was inhibited. Interestingly, HMF annealing promoted the nucleation of the α-(Fe,Co) phase. These effects are beneficial in the production of high quality nanocomposite soft magnetic materials.

## 2. Materials and Methods

The ingots used in this study were made of materials with a purity greater than 99.9 wt %. The master ingots, with the nominal composition of [(Fe_0.5_Co_0.5_)_0.75_B_0.2_Si_0.05_]_96_Nb_4_ in atom %, were prepared via arc melting under a Ti-gettered argon atmosphere. Amorphous ribbons with a cross-section of 4 mm × 100 μm were produced in a single-wheel, melt-spinning apparatus at a spinning speed of 35 m/s. The crystallographic structure and homogeneity of the ribbon were examined via X-ray diffraction (XRD, Cu Kα). Differential scanning calorimeter (DSC) measurements were carried out under a purified Ar atmosphere in a TA MDSC Q100 (TA Instruments, New Castle, DE, USA). The heat of crystallization Δ*H_x_* for the glass transition was determined by integrating the area under the DSC curve. The microstructure of the annealed sample was examined via transmission electron microscopy (TEM, Tecnai G^2^ F20, FEI, Hillsboro, OR, USA). Annealing was performed in a vacuum furnace mounted within a superconducting magnet (JMTD-12 T100, JASTEC, Tokyo, Japan) with magnetic fields of 0 T (no HMF) and 12 T. This field was well above the saturation magnetization of all the phases formed during the heat treatment. In the annealing process under 12 T, the field was applied parallel to the ribbon plane. All samples were heated to annealing temperature with a heating rate of 5 K/min and then kept at annealing temperature for 60 min. The annealed samples were furnace-cooled to below 373 K. A vibrating sample magnetometer (VSM, Lakeshore 7407, Columbus, OH, USA) was used to measure the magnetic properties of as-spun and annealed samples.

## 3. Results and Discussion

Given a heating rate of 5 K/min (the designated heat rate for the furnace in our magnet), a preliminary DSC experiment was done on a sample of as-melt-spun (AMS) [(Fe_0.5_Co_0.5_)_0.75_B_0.2_Si_0.05_]_96_Nb_4_ BMG. The glass transition temperature (*T*_g_) and the onset temperatures of primary (*T*_x1_) and secondary (*T*_x2_) crystallization were found to be 796 K, 825 K, and 966 K, respectively. The peak temperature (*T*_p_) in the DSC curve for primary phase transformation was near 835 K.

In order to determine what happens at various key temperatures, we subjected AMS samples to annealing for 60 min at *T*_g_ (796 K), at a temperature above the first peak temperature (843 K), and at a temperature identified by DSC as that of the completion of primary phase transformation temperature (883 K). Six samples in all were annealed, two at each temperature, one under 12 T HMF, and one under a zero external field.

**Figure 1:**
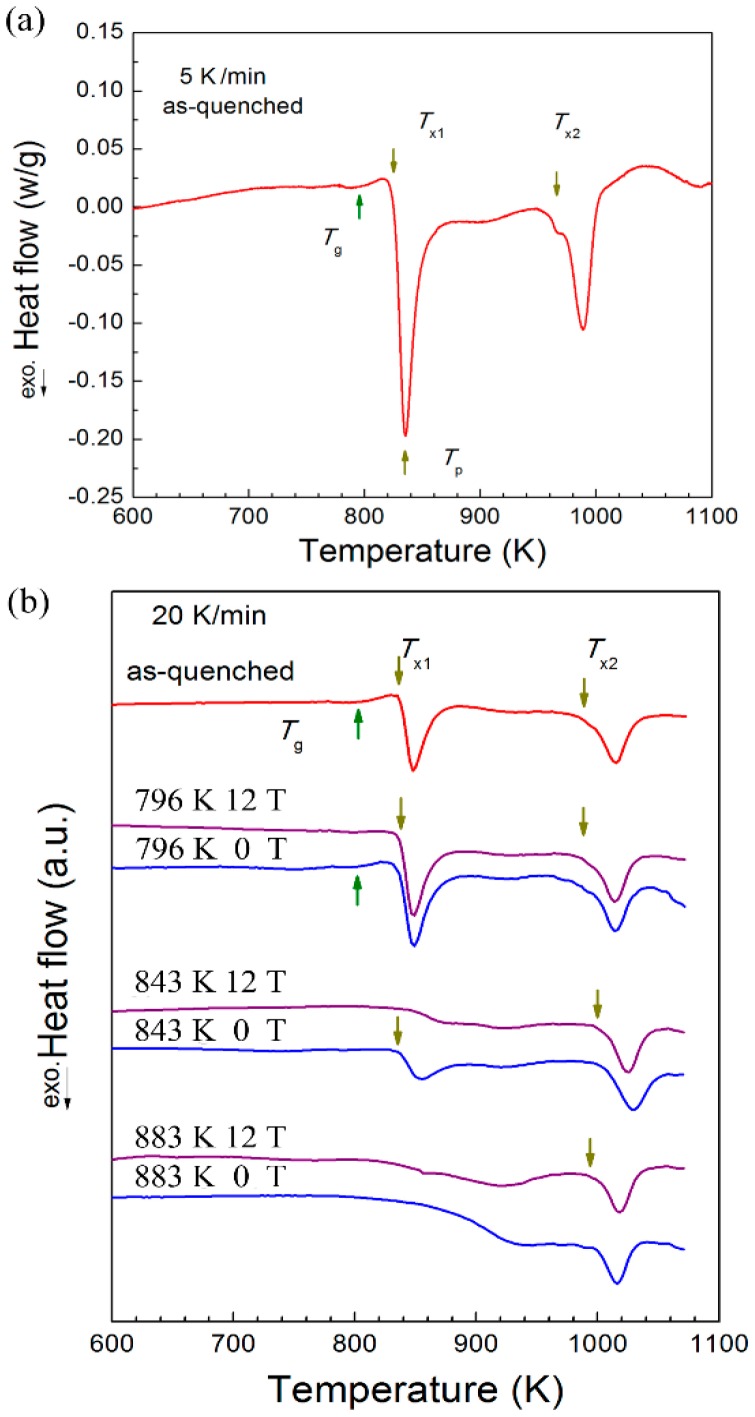
Differential scanning calorimeter (DSC) curves of the (**a**) as-melt-spun (AMS) sample with heating rate 5 K/min and (**b**) annealed samples with heating rate 20 K/min for the [(Fe_0.5_Co_0.5_)_0.75_B_0.2_Si_0.05_]_96_Nb_4_ bulk metallic glass (BMG) forming alloy. Glass transition temperature (*T*_g_) and onset temperatures of primary (*T*_x1_) and secondary crystallization (*T*_x2_) are labeled by arrows in the plots.

Once annealing was complete, we used DSC to probe the phase transitions in each sample and an AMS sample at a heating rate of 20 K/min so that we could compare the sample annealed under zero and 12 T external high magnetic field (Figure 1b), using the AMS sample as a control. The resulting DSC curve of the AMS sample exhibits the endothermic characteristics of a glass transition followed by two exothermic events that correspond to two additional phase transitions. This curve is similar to that of the original AMS sample subjected to a heating rate of 5 K/min. Thus, we concluded that the faster heating rate of 20 K/min can detect the same phase transitions as the slow rate of 5 K/min. The calculated heat energies corresponding to the primary and secondary phase transitions are 57.9 and 43.2 J/g (see Table 1).

The sample annealed for one hour at 796 K without a magnetic field shows almost the same exothermic glass transition phenomenon and amount of heat as the one that had not been annealed at all. Under the HMF, however, the DSC curve (Figure 1b) shows no obvious glass transition signal and 7.4 J/g less exothermic heat (50.5 J/g) during the primary phase transition (see Table 1). When annealed at 843 K, which is slightly higher than the primary phase transformation peak temperature, samples under a 12 T magnetic field showed almost no primary exothermic event in the DSC curve, indicating that primary phase transformation had already been completed during annealing at 843 K under the HMF before DSC tests. In contrast, the DSC curve of a sample annealed without HMF showed a primary exothermic event with a calculated heat of 21.6 J/g, which is greater than the HMF sample but less than both the original AMS and the 796 K annealed samples.

Clearly, at both 796 K and 843 K, the HMF treatment stimulated phase transformation; however, at 883 K, at which the primary phase transformation was complete, HMF annealing showed no significant impact on DSC curves. In the temperature range between the first and second crystallization temperatures, the HMF had no effect on the crystallization process because no crystalline formed. When one increased the annealing temperature to above the Curie temperature of the crystalline phases, the effect of the HMF diminished. In other words, there is a critical temperature above which HMF loses effectiveness.

**Figure 2:**
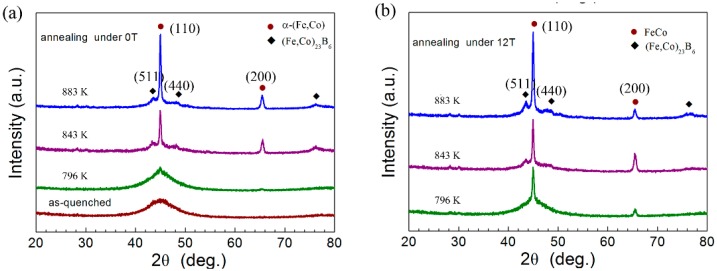
X-ray diffraction (XRD) patterns after annealing for 60 min at selected temperatures under (**a**) 0 T and (**b**) 12 T magnetic fields for [(Fe_0.5_Co_0.5_)_0.75_B_0.2_Si_0.05_]_96_Nb_4_ BMG forming alloy. The annealing temperatures are indicated above the corresponding XRD profiles.

We identified different phases in samples annealed at 796 K, 843 K, and 883 K for 60 min under 0 T and 12 T magnetic fields (see Figure 2). After annealing at 796 K for one hour without a magnetic field, the X-ray diffraction (XRD) pattern (Figure 2a) showed almost no phase change compared with the AMS sample. The XRD data is consistent with previous DSC data (see Figure 1b and Table 1), indicating the structure in the 796 K annealed sample remains the same as the AMS sample and few crystalline phase precipitants from the amorphous matrix. After annealing at 843 K and 883 K, samples show sharp Bragg peaks from both α-(Fe,Co) and (Fe,Co)_23_B_6_ crystals. The data demonstrates that two crystalline phases form simultaneously during the primary phase transition. Our result is different from that of previous researchers who found that only the Fe_23_B_6_-type phase formed in the primary phase transformation for an alloy with a near identical composition to ours [24]. Other researchers also founds discrepancies; some found only one crystal phase (Fe_23_B_6_-type phase), while others found two (α-Fe and Fe_23_B_6_-type phase) in FeNbB amorphous alloys [25,26].

When annealed at 796 K for one hour under a 12 T magnetic field, samples show sharp Bragg peaks of only the α-(Fe,Co) phase, as seen in Figure 2b. The Bragg peaks corresponding to (Fe,Co)_23_B_6_ intemetallics are not visible in the XRD pattern. The results demonstrate that the HMF applied during the annealing process significantly promotes the crystallization of the α-(Fe,Co) phase, as shown in Figure 2. However, such an effect on crystallization of the (Fe,Co)_23_B_6_ phase was marginal. Only when annealing temperature increases to 843 K under the HMF do (Fe,Co)_23_B_6_ type intemetallics subsequently form in the amorphous matrix, as shown in Figure 2b. Therefore, HMF annealing changes the crystallization sequence of the α-(Fe,Co) and (Fe,Co)_23_B_6_ phases and inhibits the co-occurrence of two phases during the primary phase transformation of a metallic glassy alloy with high boron content. This provides us with a novel way of separating the preferred α-Fe (or α-(Fe,Co)) phase from the subsequent intermetallic phases. Previously, a nanocrystalline soft magnetic composite with only the α-(Fe,Co) or α-Fe_3_Si phase was made via the addition of Cu to the alloys with a boron content value lower than 20 atom % [6,27].

**Figure 3:**
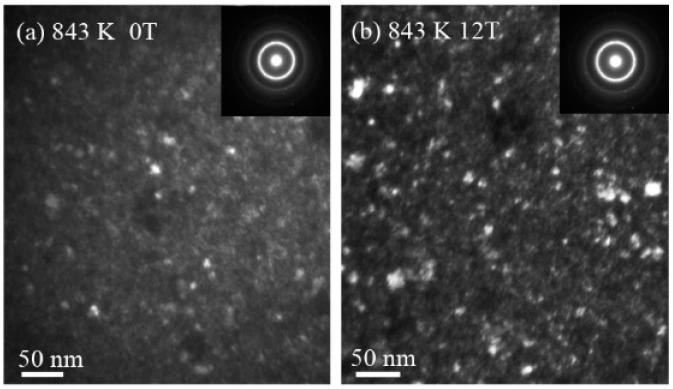

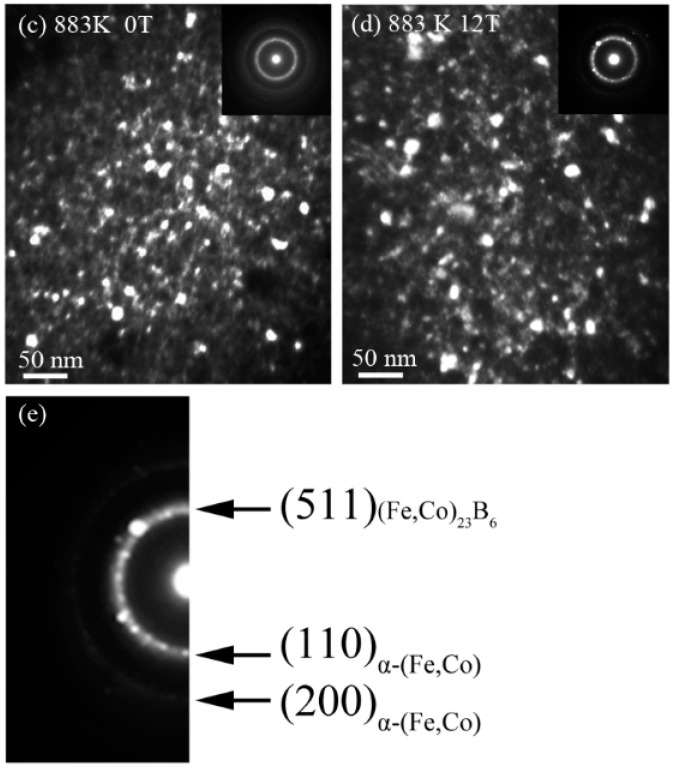
Dark-field transmission electron microscopy (TEM) micrographs and diffraction patterns for [(Fe_0.5_Co_0.5_)_0.75_B_0.2_Si_0.05_]_96_Nb_4_ metallic glassy sample annealed at 843 K and 883 K for 60 min—(**a**,**c**) with 0 T; (**b**,**d**) 0 T, respectively; (**e**) is the characterization of the diffraction patterns of the inset image in (**d**).

Figure 3 shows the dark-field images and the corresponding selected-area electron diffraction images (SAED, the inset images) from the annealed samples. Due to the low volume fraction of crystalline for alloys annealed at 796 K, only TEM results of the sample annealed at 843 K and 883 K are given in Figure 3a–d, respectively. Two high intensity diffraction rings are from (110) and (200) of the α-(Fe,Co) phase, and the first weak ring and spots with a lattice parameter greater than (110) of α-(Fe,Co) are from (511) of the (Fe,Co)_23_B_6_ phase, as shown in Figure 3e. Dark filed images taken from the first ring are shown in Figure 3a–d, respectively. The TEM dark-field images reveal that spherical nanocrystals (bright ones) are uniformly dispersed in the amorphous matrix as shown in Figure 3a,b for samples annealed at 843 K under 0 T and 12 T magnetic fields, respectively. The volume fraction of crystalline for samples annealed at 843 K under 12 T is greater than that annealed under 0 T. This conclusion is consistent with the one obtained from DSC and XRD results in Figure 1b and Figure 2, confirming that the 12 T HMF promotes the primary crystallization of the [(Fe_0.5_Co_0.5_)_0.75_B_0.2_Si_0.05_]_96_Nb_4_ BMG alloy. When annealed at 883 K, the images show little difference in the volume fraction of the α-(Fe,Co) crystalline phase, while the size of the nanocrystallines increases from 7 ± 2 nm to 9 ± 2 nm when HMF increases from 0 T to 12 T. This indicates that the HMF promotes the growth of the crystalline.

The saturation magnetization (*M*_s_) and coercivity (*H*_c_) of the as-cast and annealed [(Fe_0.5_Co_0.5_)_0.75_B_0.2_Si_0.05_]_96_Nb_4_ sample are shown in Table 1. Generally speaking, since the HMF treatment can promote the primary phase transformation, the *M*_s_ of the samples annealed at 796 K and 843 K under 12 T are 134.4 emu/g and 133.3 emu/g, higher than the values of the samples treated without a HMF. The promotion of α-(Fe,Co) phase annealed at 796 K under 12 T helps the reduction of *H*_c_ because more exchange coupling takes place. However, due to the higher amount of crystallizations of the (Fe,Co)_23_B_6_ phase and the low residual amorphous matrix in the annealed samples annealed at 843 K under 12 T, which cause more interfaces to appear, blocking the domain wall motions, the *H*_c_, compared with the samples annealed without a magnetic field, is increased.

**Figure 4:**
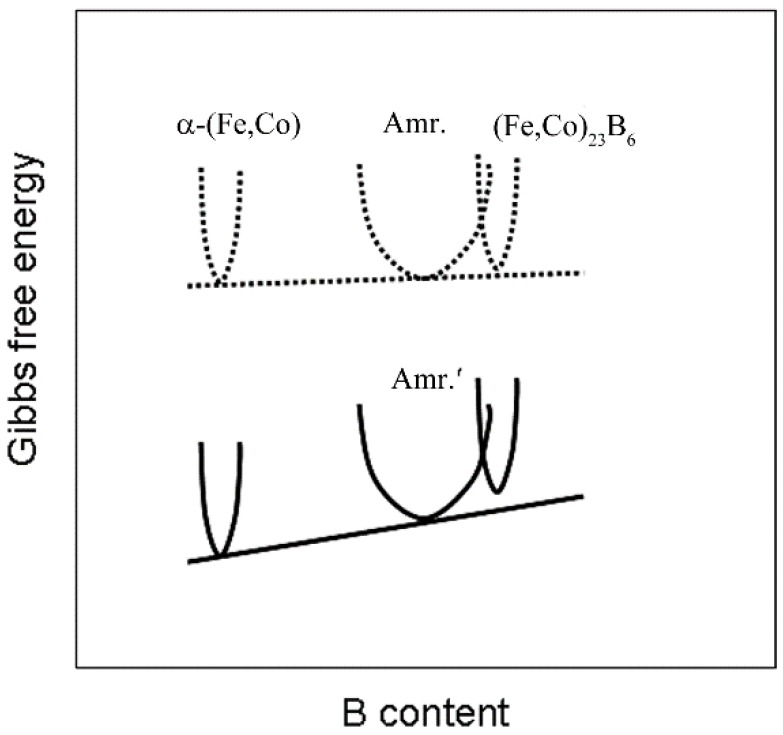
Schematic diagram of Gibbs free energy vs. boron concentration for α-(Fe,Co), the amorphous matrix, and (Fe,Co)_23_B_6_ without (dash line) and with (solid line) 12 T magnetic field.

The above results indicate that the primary phase transformation process was facilitated by the 12 T magnetic field during the annealing process in the investigated temperature ranges for the [(Fe_0.5_Co_0.5_)_0.75_B_0.2_Si_0.05_]_96_Nb_4_ BMG alloy with high boron content. The α-(Fe,Co) phase forms prior to the (Fe,Co)_23_B_6_ phase when samples are annealed at a glass transition temperature under a 12 T magnetic field. The influence of the HMF on the crystallization sequence for the [(Fe_0.5_Co_0.5_)_0.75_B_0.2_Si_0.05_]_96_Nb_4_ BMG alloy is deduced by changing the Gibbs free energies of the α-(Fe,Co) and (Fe,Co)_23_B_6_ phases at the annealing temperature, 796 K. A similar effect of the HMF has been observed on carbide precipitation during the annealing of an Fe–C alloy under a HMF [15,19]. As we all know, the introduction of a HMF changes the phase equilibrium, which is beneficial to the nucleation of the ferromagnetic phase [19,28]. The magnetic energy contribution part is simply expressed as: $-\mu_{0}M•H$, where $\mu_{0}$ is the permeability of free space, *M* is the magnetization, and *H* is the applied field. In our case, because *H* is significantly higher than *M* and saturation magnetization, a large Gibbs energy change was introduced. However, the reduced Gibbs energy due to the increased magnetic field is higher when *M* is below *M*_s_ because both *H* and *M* can be increased. For the ferromagnetic phase above saturation magnetization, the energy contribution is in direct proportion to the product of *M*_s_ and *H*. The $Ms_{\alpha -FeCo}$ is higher than $Ms_{Fe_{23}B_{6}}$ for the α-(Fe,Co) phase with more ferromagnetic atoms per mole, which provides a greater overall magnetic moment of the phase [19]. Thus, when the 12 T HMF is applied, the Gibbs free energy of α-(Fe,Co) is lower than that of the (Fe,Co)_23_B_6_ phase. Therefore, the precipitation priority of α-(Fe,Co) is promoted in contrast to the (Fe,Co)_23_B_6_ phase. The phase-equilibrium dependence of the magnetic field can be illustrated schematically in Figure 4, which qualitatively shows the Gibbs free energy changes for the different phases with respect to boron content in the alloy under the HMF. B content is just a representative of the studied alloy. Annealing at 796 K without a HMF results in the coexistence of three phases: the α-(Fe,Co), the amorphous, and the (Fe,Co)_23_B_6_ phases. Annealing under a HMF at the same temperature results in the coexistence of the α-(Fe,Co) and remaining amorphous without boride. It is assumed that the deduced Gibbs free energy of the α-(Fe,Co) phase is larger than boride due to the lower B content and higher Fe content in HMF annealing.

Except for the magnetization energy, the magnetization process may also be affected during the crystallization under the HMF and hence the magnetization process may influence the crystallization sequence. The formation of the (Fe,Co)_23_B_6_ phase in the crystallization process requires a large extent of atomic rearrangement in the amorphous matrix to form both the Fe–B and Co–B bonds. The electrons can transfer from metalloid B to the d-shells of transition metals Fe and Co, which creates strong s–d or p–d hybridization, as shown with the CoNbB alloy [29]. During HMF annealing, in comparison with the α-(Fe,Co) phase, the hybridization will be suppressed and prevent boride formation.

## 4. Conclusions

When applied in combination with the adjustment of annealing temperatures, a HMF is a versatile tool for fabricating magnetic nanocomposite materials from parent amorphous alloys. In order to enhance soft magnetic properties, the introduction of a HMF can separate the α-(Fe,Co) phase from the subsequent intermetallic boride phase solely by changing the crystallization sequence, without the addition of any other alloying elements. This method is valid even for alloys with high B content. At the beginning of the first crystallization annealing process, the HMF largely improves the formation of the α-(Fe,Co) phase but has marginally effects on borides, which helps to produce the composite with only α-(Fe,Co) nanocrystalline in the amorphous matrix without any boride. The mechanism of the HMF changing the crystallization sequence can be ascribed into the reduced Gibbs free energies and the suppressed s–d or p–d hybridization for ferromagnetic phases with the respective different magnetization.

## Figures and Tables

**Table 1:** The heat of crystallization, magnetic properties, and phase formation for the [(Fe_0.5_Co_0.5_)_0.75_B_0.2_Si_0.05_]_96_Nb_4_ metallic glassy sample upon annealing at different conditions for 1 h.

Annealing Condition		Δ*H*_1_ (J/g)	Δ*H*_2_ (J/g)	*M_s_* (emu/g)	*H*_c_ (A/m)	Phase
AMS		57.9 ± 0.1	43.2 ± 0.1	133.2 ± 0.2	7.1 ± 0.1	Amr.
796 K	12 T	50.5 ± 0.1	41.1 ± 0.1	134.4 ± 0.4	1.5 ± 0.2	Amr. + α-(Fe,Co)
	0 T	57.9 ± 0.1	40.1 ± 0.1	132.8 ± 0.5	3.3 ± 0.1	Amr. + α-(Fe,Co) + (Fe,Co)_23_B_6_
843 K	12 T	0	39.7 ± 0.1	133.3 ± 0.3	53.8 ± 0.4	Amr. + α-(Fe,Co) + (Fe,Co)_23_B_6_
	0 T	21.6 ± 0.2	40.0 ± 0.1	130.7 ± 0.2	26.5 ± 0.3	Amr. + α-(Fe,Co) + (Fe,Co)_23_B_6_
883 K	12 T	0	35.5 ± 0.1	129.8 ± 0.2	56.5 ± 0.5	Amr. + α-(Fe,Co) + (Fe,Co)_23_B_6_
	0 T	0	33.9 ± 0.1	133.2 ± 0.3	54.1 ± 0.4	Amr. + α-(Fe,Co) + (Fe,Co)_23_B_6_
